# A Multidimensional Approach to Talent Identification in Youth Volleyball through Declarative Tactical Knowledge and Functional Fitness

**DOI:** 10.3390/jfmk9010029

**Published:** 2024-02-06

**Authors:** Francesco Sgrò, Antonella Quinto, Mario Lipoma, David Stodden

**Affiliations:** 1 KG4SPA—Kore Research Group for Sport and Physical Fitness Assessment, 94100 Enna, Italy; antonella.quinto@unikore.it (A.Q.); mario.lipoma@unikore.it (M.L.); 2 Department of Human and Society Sciences, University of Enna “Kore”, 94100 Enna, Italy; 3 Department of Educational and Developmental Science, University of South Carolina, Columbia, SC 29208, USA; stodden@mailbox.sc.edu

**Keywords:** functional motor competence, musculoskeletal fitness, net games, perceptuomotor coordination

## Abstract

This study aimed to assess which multidimensional performance indexes were the best predictors of talent identification in volleyball. Fifty-five female players (age: 13.8 ± 1.81 years; mass: 55.12 ± 8.12 kg; height: 158.23 ± 7.62 cm) were clustered into two groups according to some physical characteristics (i.e., the first group included players with more favorable performance predictors). Musculoskeletal Fitness (MSF), Functional Motor Competence (FMC), and Declarative Tactical Knowledge (DTK) were measured as multidimensional indexes of performance. Moderate-to-large differences between groups were found for each index in favor of the first group. Regression analyses were performed to examine the variance explained by MSF, FMC, and DTK in the two groups. A model with FMC components explained slightly more variance in the group predictor variables (*R*^2^ = 0.53) than a model using only MSF components (*R*^2^ = 0.45). Among FMC components, the score of the Throw-and-Catch test resulted in the best predictor (Odds Ratio = 1.58) for determining group selection, followed by the score of the Supine-to-Stand-and-Go test (Odds Ratio = 0.02). An additional model composed by MSF and FMC significant predictors (i.e., functional fitness index) and DTK explained 63% of the variance (*R*^2^ = 0.63), and these were significant predictors of group membership (Odds Ratio = 6.32 and Odds Ratio = 1.51, respectively). A more comprehensive multidimensional analysis of youth performances is warranted to identify and monitor the best players in a youth volleyball context.

## 1. Introduction

Volleyball is a dynamic and challenging sport that requires a combination of physical and tactical skills. As an open-skill sport, it is characterized by a dynamic environment that requires high perceptuomotor and cognitive demands during a game [1,2]. The identification of higher-level players has been traditionally influenced by height, which is an attribute that offers advantages in this sport [3], in combination with technical abilities and physical performance characteristics [4,5]. Explosive skills such as jumping and striking are also identified as key factors that contribute to success during elite competitions [6,7]. The repetitive nature of explosive skills performed in volleyball matches requires an adequate foundation of Musculoskeletal Fitness (MSF), which includes cardiorespiratory endurance, muscular strength, muscular endurance, and muscular power [8]. For example, neuromuscular mechanisms (e.g., stretch-shortening cycle performance and high motor unit recruitment levels) are fundamental aspects of muscular strength and power that are crucial to various unilateral and bilateral jumps performed by elite volleyball athletes [9]. At the same time, a key role in expert performance is noted by eye–hand coordination, multijoint coordination, perceptuomotor coordination, and speed of movement [3,10,11,12,13,14]. These capabilities are inherently demonstrated in the assessment of Functional Motor Competence (FMC) skills and are key elements of the perceptuomotor integration process needed to perform goal-direct movements [15,16,17]. Previous studies suggested addressing how several performance-related factors (e.g., MSF and FMC), together as a multicomponent concept (i.e., Functional Fitness), impact on talent identification in sport [18,19]. For this reason, evaluating MSF and FMC could offer a complete and detailed perspective on the functional fitness of volleyball players.

Moreover, volleyball is a team sport that requires strong collaboration and coordination between teammates. Therefore, players also must be able to integrate communication, coordinate tactical movement strategies with teammates, and understand the dynamics of the opponent’s strategies. However, if the execution of gameplay tactics is not adequate, functional fitness characteristics may be not sufficient to promote high-level performance. For this reason, it is imperative to adopt a broader and multidimensional approach in the selection of volleyball players that takes into account the levels of tactical knowledge of players in addition to their functional fitness. Tactical knowledge is defined as the ability to identify problems that arise while a game is in progress and to select the skills needed to solve them [20]. In sports, good tactical knowledge allows experienced athletes to effectively identify, remember, and manipulate the information contained in memory [15] to make effective decisions in a dynamic environment. In this sense, elite athletes continually make rapid and precise decisions during performance [21,22,23,24,25]. In addition, elite athletes are distinguished from beginners by their greater declarative and procedural tactical knowledge [26,27,28].

As noted in a previous study [29], Declarative Tactical Knowledge (DTK) is the knowledge of factual information related to performance. It coincides with “knowing what to do” during the game based on knowledge of the rules, positional requirements, and offensive and defensive strategies. In addition, Procedural Tactical Knowledge (PTK) is intimately tied to the authentic dimension of the game and is identified with “know how to do”, which is the ability of a player to effectively execute tactics and understand the dynamics of the game [30]. Anderson [31] highlighted the interconnection between these forms of knowledge during the learning process of skills. Initially, individuals consciously acquire rules, concepts, and gameplay characteristics. As learning progresses, the solutions found to solve tactical problems become strengthened and automatic. This phenomenon represents an automatization of behavior where the acquired knowledge guides the action without requiring a conscious reflection. For this reason, it is advisable to focus on DTK levels, especially in novice athletes, to predict future procedural behavior [32].

Based on the need to integrate physical performance characteristics with tactical knowledge variables, we suggest that a multidimensional approach for talent identification in youth sports is needed, specifically in the early stages of the sport specialization phase (age 11–15 years) [4,33,34]. Current studies related to talent-identification strategies and the selection process in youth volleyball focus on either physical development aspects (e.g., body height) or technical skills (e.g., serving) [18]. However, no research has integrated tactical knowledge components and their interaction with critical physical performance dimensions needed in volleyball. 

In this respect, in this study we have initially determined two groups of athletes (i.e., first and second) by means of a clustering approach which used players’ height, BMI, and lower-body muscular strength/power characteristics as a predefined set of performance predictors (as proposed in a previous study [4]). The first group included players who demonstrated more favorable performance predictors. Then, we addressed how DTK, MSF, and FMC components predict the odds that a youth volleyball player could be assigned to one of the previous groups. Therefore, the study aimed to assess which multidimensional performance indexes were the best predictors of talent identification in volleyball. In this respect, we hypothesized that higher scores in DTK, FMC, and MSF would all independently predict group membership. Lastly, we hypothesized the collective integration of FMC and MSF components and DTK levels would better explain group membership of athletes.

## 2. Materials and Methods

### 2.1. Participants

Fifty-five female junior volleyball players (age: 13.8 ± 1.81 years; mass: 55.12 ± 8.12 kg; height: 158.23 ± 7.62 cm) took part in this study. Girls played in the two different teams of a city located in southern Italy. Inclusion criteria used to sample the participants included that they were free of musculoskeletal injuries in the two weeks before the study, they did not take any nutritional supplements, and they had at least one year of practice in volleyball. Because the girls’ age was lower than 18 years old, parents or legal guardians were informed about the characteristics of the study through informed consent to allow participation in the research. The methodological procedure used in this study followed the indications of the Declaration of Helsinki [35] and the Ethics Committee of the University of Enna approved this study (Prot. No. 14006-2023).

### 2.2. Measures

#### 2.2.1. Anthropometric Data and Group Predictor Variables

Body weight and height were measured using an electronic scale (Sensit Smart Scale, Prozis, Esposende, Portugal) and a stadiometer, and were expressed in kilograms and centimeters, respectively. Body Mass Index (BMI) was calculated by using the formula body weight/body height^2^ and was expressed in kg/m^2^. Lower body muscular strength and power was assessed trough Standing Long Jump (SLJ). The distance from the starting line to the landing position of the feet was recorded to the nearest 0.01 cm. If the feet were not aligned at the landing, the distance was estimated by the backmost foot. The three best scores of a total of seven trials were averaged and used for further analysis. The validity of this test has been verified in previous studies [36].

#### 2.2.2. Declarative Tactical Knowledge

The questionnaire used to assess Declarative Tactical Knowledge was composed of 30 questions, selected from a large sport-specific dataset [37]. Each question accounted for only one correct response among the three provided options. A point was assigned only for each correct response, with a best possible score of 30. To ensure content validity, the questionnaire was arranged according to the indications provided in a previous study [37]. Specifically, questions were selected to respect the following topic-related percentage: technique (40%), rules and score (20%), game strategy (20%), safety and equipment (8%), and sport-specific terminology (12%). Because some questions related to rules and scores were out of context with respect to the actual volleyball system, some adaptations were made to make it up-to-date when necessary. The reliability of the questionnaire was preliminarily verified using the Kuder–Richardson 21 formula [38]. The questionnaire is available upon request to the corresponding author of the study.

#### 2.2.3. Musculoskeletal Fitness

The MSF assessments were conducted following FitnessGram protocols for Progressive Aerobic Cardiovascular Endurance Run (PACER) and the 90° Push-up test [39,40]; additional tests to assess grip strength and core muscular endurance were also included. The PACER was used to assess cardiorespiratory endurance. The objective of the test was to complete adequately (i.e., by crossing the end line in time) as many laps of 20 m to a graduated pace as possible. The test was stopped when an athlete failed to complete a lap according to the specified pace for the second time. The number of laps run by each athlete was counted and used for further analysis. The 90° Push-Up permitted the assessment of upper-body muscular endurance. The objective of the test was to perform as many push-ups as possible while keeping a specified pace punctuated by a digital metronome (i.e., up–down). The number of completed push-ups was recorded and used for further analysis. Validity and reliability of these tests were assessed in previous studies [39,41]. A Jamar Hydraulic Hand Dynamometer was used to measure upper extremity isometric strength [40]. The measure was recorded three times on each hand to the nearest 0.1 kg. The best scores registered for each hand were averaged and used for further analysis. Plank exercises were administered to assess core muscular endurance [42]. The objective of the test was to maintain an adequate horizontal posture as long as possible. The time was stopped when an athlete lost proper form or when her knees/stomach touched the ground. The time of the trial was recorded using a digital stopwatch nearest 0.01 s and used for further analysis. Previous research has demonstrated acceptable validity and reliability for these tests [43,44].

#### 2.2.4. Functional Motor Competence

The FMC assessment was conducted using three tests. Throw-and Catch (T&C) and Kick-and-Receive (K&R) tests were used to evaluate object projection and reception skill, as well as perceptuomotor integration. The Supine-to-Stand-and-Go (STS&Go) test was used for assessing “righting abilities” such as stability, control, and coordination of the center of mass [45] and speed and agility. T&C involved throwing and catching a standard tennis ball against a wall at least six meters high within 30 s [14] Participants stood at least three times their height away from the wall, marked by a line on the floor. Both feet had to be behind the line for a valid throw-and-catch performance. No specific throwing technique was mandated, while only the use of hand(s) was allowed to catch the ball. Participants had a brief practice (i.e., at a maximum of ten T&Cs) before two 30 s trials, and the highest score recorded in these trials was used for further analysis. Developmental validity of this test was previously assessed [14]. In K&R, participants kick a standard soccer ball against a solid wall and receive it (kickback) as many times as possible in 30 s behind a kick line at least three times the participant’s height away from the wall. No specific kick technique is mandated, allowing players to use their preferred foot and style. The ball can be kicked again directly or after being stopped with any part of the body except the hands and arms. Players had a brief familiarization period (i.e., at a maximum of ten K&Rs) before two 30 s trials, and the highest score recorded in these trials was used for further analysis. Finally, during STS&Go, participants had to stand up as fast as possible, sprint for 10 m, circle a cone, and sprint back through the starting point as fast as possible. Participants started from a supine position, with their palms up and their feet behind a fixed starting line. Each participant performed two trials and the rest between trials was 30 s. Research staff used a stopwatch to record the time elapsed between the starting command and the instant when the player crosses the starting line on the way back. The time was recorded to the nearest 0.01 s and the best score of these two trials was used for further analysis. 

The research team, who directly worked in the data collection procedure related to this study, was composed of a senior researcher in sport pedagogy, a doctoral student in physical activity for health and wellbeing, and seven graduate students in sport science. The senior researcher trained the other members of the team for one month before beginning the data collection procedure. The scores recorded by the senior researcher and each staff member during two practical sessions of test administration were used to test inter-rater reliability. The inter-rater score ranged from 0.95 to 0.99 for the nine tests administered on the first day and from 0.94 to 0.99 on the second day.

### 2.3. Procedures

The administration of tests and questionnaire was performed in the gym used by the volleyball teams for their training sessions. The tests were performed on two different days, one day for each team, and each session lasted three hours at maximum. Before the MSF and FMC tests began, players performed a 15-min-long general warm-up to activate their bodies and increase their baseline cardiac frequency. Then, players were divided into small groups (three or four players per group) and started the tests. The first test performed by the participants was the PACER and it was the only one performed by multiple players (five at maximum) at the same time. Then, the gym was organized into multiple stations to permit the administration of other tests at the same time. Each group moved from one station to the other without a pre-scheduled order, thus, providing a general randomization of test implementation. At the end of tests, participants filled in the questionnaire to assess their DTK using their smartphone. The time for filling in the questionnaire was 25 min on average. 

### 2.4. Data Analysis

Data were preliminarily checked for accuracy, missing values, and outliers. Then, they were assessed to address assumptions of linearity, independence of errors, and multicollinearity. A non-hierarchical k-means cluster method, with the number of clusters fixed at two, was used to group participants into two groups (i.e., first and second) according to group predictor variables.

Descriptive statistics for anthropometric data and group predictor variables, DTK, and scores of FMC and MSF tests were calculated, and stratified by the aforementioned group selection membership. Independent *t*-tests were performed to assess differences between groups for each measure. For each comparison, we reported mean differences and effect size measure (i.e., Cohen’s *d*) with their 95% confidence interval, respectively. Four different logistic regression analyses were performed to provide the most parsimonious model for predicting group membership (i.e., the second group was used as the baseline). The first model accounted for the DTK questionnaire score; the second accounted for MSF variables; the third accounted for the FMC variables; the fourth accounted for a composite functional fitness index (FFIndex), estimated by summing the scores of resultant significant predictors in the previous MSF and FMC models. Because the measuring scales of MSF and FMC tests were different, their data were preliminarily converted into a z-score. Furthermore, because the score of the STS&Go test had a reversed interpretation with respect to the others (i.e., decreased time = better performance), the relevant data were multiplied by −1. For each regression model, we reported Akaike Information Criterion (AIC), deviance (χ^2^), and Cox-Snell *R*^2^ summary statistics in addition to unstandardized beta coefficients of each significant predictor(s) and the related Odds Ratio (OR). AIC and *R*^2^ were used to assess the goodness of a model to fit the outcome and were interpreted as follows: lower AIC values and higher *R*^2^ corresponded to a better model fit. Cut-offs used to interpret Cohen’s *d* were: 0.2 = small, 0.5 = medium, 0.8 = large. The analyses were performed using R Statistical Software for Mac OS X (version 4.0.3—R Core Team, 2023) and the alpha test was set to 0.05.

## 3. Results

Descriptive statistics for anthropometric characteristics and predictor variables, scores of functional fitness tests, and DTK scores, stratified by the forecited groups, are shown in Table 1. Assumptions of normal data were initially verified; therefore, the previously noted analyses were conducted. Three participants were unable to complete all the tests and they were excluded from further analyses. The number of participants per group and the mean values of the parameters used to build these groups by means of the k-means cluster approach are described in the following points:

1. First group: n = 28; body height = 161.82 cm; BMI: 21.70 kg/m^2^; SLJ = 175.52 cm
2. Second group: n = 24; body height = 153.61 cm; BMI: 22.64 kg/m^2^; SLJ = 133.96 cm

For each test, the scores of players belonging to the first group were better than the ones of the players of the second group (see Table 1). The effect of these differences was medium for Push-ups (0.64) and K&R (0.65) and large for PACER (1.07), Handgrip (1.23), Plank (1.11), T&C (0.92), and STS&Go (−1.79). 

Regression analyses were performed to assess the effect of four different models (i.e., based on DTK, MSF, and FMC variables) on the likelihood of athletes to be included into the first group.

Table 2 and Table 3 show the global characteristics and the significant coefficients for each model, respectively.

According to their χ^2^ values and *p*-values, all the models were statistically significant. The DTK model explained 31% (i.e., Cox-Snell *R*^2^) of the variance of group association. MSF and FMC models explained 45% and 51% (i.e., Cox-Snell *R*^2^) of the variance of group association, respectively. The last model, which included the FFIndex (MSF and FMC) and DTK, provided the best model fit (i.e., AIC value was the lowest and *R*^2^ the higher) and explained the most variance of group association (Cox-Snell *R*^2^ = 0.63).

## 4. Discussion

The study aimed to explore whether analysis with the FMC and MSF components along with DTK levels provides a better explanation for a volleyball players’ group membership with more favorable performance predictors. In this respect, players were initially grouped into two groups (i.e., first and second) following the procedures and the performance predictors proposed in a previous study [4]. Then, DTK and MSF and FMC indexes were used to assess differences between groups and predict the membership of players in performance groups. The integration of DTK and certain MSF and FMC measures (i.e., Plank, Handgrip, T&C, and STS&Go) demonstrated the best goodness-of-fit for predicting membership. Therefore, the use of multifactorial profiles may be an important step forward as an approach to identify athlete talent in a youth sport context.

The highest effect size measures related to between-group differences were registered for the scores of STS&Go (Cohen’s *d*: 1.79), Plank (Cohen’s *d*: 1.11), and Handgrip (Cohen’s *d*: 1.23) tests and the DTK questionnaire (Cohen’s *d*: 1.36). This result seems to underline that the differences between players’ levels of performance also depend on aspects related to physical and tactical perspectives. As research on the identification of talent in youth volleyball players through a multidimensional approach is rare, the current results could be considered as novel findings and provide several implications for grassroots coaches and, as a consequence, for the talent-identification process.

To better understand how these performance indexes, alone or combined, could predict the membership to the first groups, four different regression models were estimated. 

In agreement with the statistics of the first model (i.e., DTK), the chance to stay in or reach the first group increases by 1.36 times for each one-unit increase in DTK score, and therefore the odds of an athlete being in the first group increased by 36% for each one-unit increase in questionnaire score. DTK is a relevant part of volleyball performance and it has been identified as predictive of the development of other aspects of cognitive performance [31]. In addition, this result is in agreement with the study of Gil and colleagues [46], who argued that the volleyball players identified as the best were characterized by the highest scores of DTK with respect to the others. 

The second perspective of our analysis assessed how different components of the functional fitness were able to predict the membership of our players to the first group. A model which included only MSF components explained 31.3% of the proportion of variance in the membership of the two groups. The odds of an athlete being in the first group increased by 1.03 times for each one-unit increase in plank time (with handgrip score holding at a fixed value) and increased by 1.30 times for each one-unit increase in handgrip score (with plank time holding at a fixed value). As indicated in a previous study [47], volleyball is an open-skill sport where several phases of gameplay require an adequate level of core stability to repeatedly perform effective total body actions. Moreover, good development of core strength helps to maintain an effective posture, which is useful to generate power with lower limbs and transfer it to the upper part of the body as demanded in some volleyball actions such as finalizing the attack or receiving the ball. Therefore, the significant predictors resulting in this model support this perspective of volleyball performance.

When only the FMC components were considered, the proportion of variance in the membership of the two groups was 51%. The odds of an athlete moving into the first group increased by 1.58 times for each one-unit increase in TC score (with STS&Go time holding at a fixed value) and increased by 0.02 times for each one-unit increase in STS&Go time (with TC holding at a fixed value). These tests assess speed, agility, hand–eye coordination, and perceptuomotor coordination. As described by the literature, all the above factors are important indicators of performance in volleyball. In detail, speed and agility are connected [48]: an agile player can change direction quickly, reach the ball in difficult positions, and perform effective movements, which is crucial to competing at a high level. For example, speed and agility are crucial to creating attacking opportunities and reacting quickly to opposite-team attacks [1,49]. Fast players can also reach appropriate tactical positions more quickly [50]. High levels of hand–eye coordination allow players to perform technical fundamentals effectively and accurately. For example, as demonstrated by Sakthivel and colleagues [51], hand–eye coordination positively impacts the volleyballer’s service skills. Perceptuomotor coordination is essential because it represents the speed and the accuracy with which a player can perceive a game situation, process information, and respond appropriately [11,52]. 

The last model accounted for the combination of the FFIndex and DTK. The FFIndex was estimated by considering the significant predictors of MSF and FMC models. In this case, the odds of being in the first group increased by 1.50 times for each one-unit increase in DTK score (with FFIndex holding at a fixed value) and increased by 6.32 times for each one-unit increase (with DTK score holding at a fixed value). According to Boichuk and colleagues [50], the balance between physical condition, functional competence, and tactical knowledge is essential to demonstrate optimal performance in adult volleyball players. In this sense, athletes who reach a good level of functional fitness and tactical skills can better adapt to different game situations. 

All the estimated models were significant, but the model which included FFIndex and DTK resulted with the best goodness-of-fit score (i.e., lowest AIC and higher *R*^2^), and therefore both our hypotheses were verified. As a consequence, the current results support the idea that the identification of sporting talent cannot and should not be limited to a single characteristic or skill. Each athlete is a unique individual with a different combination of talents. However, as far as the authors are aware, no study has investigated the levels of Declarative Tactical Knowledge (DTK) as a talent-identification factor for volleyball players. In this sense, this study has confirmed the importance of the athlete’s physical performance characteristics to aspire to the highest levels, but underlines the need to improve this perspective of performance assessment with that relating to DTK. 

The results provided in this study have the following limitations. The assessments of FMC and MSF were developed out of gameplay flow, and therefore it is not possible to accurately identify how players could translate their fitness skills into technical performance. In addition, the current results did not account for biological age and/or relative age effect of players. Third, the DTK accounts for a partial representation of the level of knowledge, and therefore the factual perspective of knowledge is not to be considered in this study. 

## 5. Conclusions

Incorporating different dimensions of performance in the identification of the best players ensures a complete and balanced assessment strategy able to identify the sporting talent accurately. In addition, a multidimensional analysis of performance offers development opportunities adapted to the specific needs of each athlete. In the sport context, it is essential to ensure that talent-identification strategies are optimized and that every young person has the opportunity to explore their abilities. The approach used in this study accounted for a multidimensional analysis of youth volleyball players’ performances through reliable and easy-to-use tests. In the current results, FMC components (i.e., speed, agility, and perceptuomotor coordination) seem able to predict the membership of athletes in the first group better than the MSF components, even if the integration of multidimensional indexes (i.e., FMC, MSF, and DTK) represents the best way to model the highest performance. By focusing on aspects such as DTK and functional fitness, coaches can identify young talents who may not have reached high level performance yet, but who exhibit exceptional capabilities in other crucial game areas. For this reason, we consider it is necessary for future research to focus even more on the multidimensional nature of sport talent, especially in volleyball, and on the need to administer feasible, effective, and reliable assessment tools that can be used to support coaches in identifying and monitoring the best talents of their club.

## Figures and Tables

**Table 1:** Descriptive statistics of anthropometric characteristics, predictor variables, DTK, MSF, and FMC tests scores stratified by groups.

	First Group (n = 28)	Second Group (n = 24)	Comparison Data			
Variables	Mean ± SD	Mean ± SD	MD	95%CI MD	ES	95%CI ES
Anthropometric data and predictor variables						
Age (y)	15.00 ± 1.36 ***	12.45 ± 1.44	2.54	1.75, 3.32	1.82	1.16, 2.46
Mass (kg)	56.89 ± 7.5	53.40 ± 8.97	3.49	1.09, 8.08		
Height (cm)	161.82 ± 5.41 ***	153.61 ± 7.85	8.21	4.50, 11.93	1.23	0.63, 1.82
BMI (kg/m^2^)	21.70 ± 2.51	22.64 ± 3.68	−0.93	−2.67, 0.98		
SLJ (cm)	175.52 ± 15.28 ***	133.96 ± 15.43	41.56	33.00, 50.12	2.71	1.95, 3.47
Knowledge component						
DTK	22.07 ± 4.23 ***	16.66 ± 3.65	5.40	3.21, 7.60	1.36	0.75, 1.96
MSF tests						
PACER (no. of laps)	24.42 ± 7.59 ***	16.37 ± 7.50	8.05	3.84, 12.27	1.07	0.48, 1.65
Handgrip (kg)	32.84 ± 3.52 ***	27.70 ± 4.81	5.13	2.73, 7.53	1.23	0.63, 1.82
Push-ups (no. of push-ups)	4.00 ± 4.37 *	1.70 ± 2.37	2.29	0.36, 4.22	0.64	0.08, 1.19
Plank (s)	98.93 ± 33.65 ***	61.62 ± 33.33	37.05	18.58, 56.02	1.11	0.52, 1.70
FMC tests						
T&C (no. of T&Cs)	13.00 ± 1.63 **	11.37 ± 1.92	1.62	0.62, 2.63	0.92	0.34, 1.48
K&R (no. of kicks)	8.28 ± 1.51 *	7.08 ± 2.18	1.20	0.13, 2.27	0.65	0.09, 1.21
STS&Go (s)	7.05 ± 0.39 ***	7.99 ± 0.66	−0.95	−1.26, −0.64	−1.79	−2.43, −1.13

Note: SD: standard deviation; MD: mean difference; 95% CI: 95% confidence interval; ES: effect size; cm: centimeters; kg: kilograms; m: meters; s: seconds; *: *p* ≤ 0.05; ** ≤0.01; *** ≤0.001 significantly different from players included in the second group.

**Table 2:** Summary of estimated logistic regression models.

Model	AIC	χ^2^	Δ*df*	*p*	Cox-Snell *R*^2^
DTK	56.79	18.99	1	<0.0001	0.31
MSF	50.50	31.28	4	<0.0001	0.45
FMC	42.34	37.43	3	<0.0001	0.51
FF + DTK	25.54	52.24	2	<0.0001	0.63

Note: AIC: Akaike Information Criterion; Δ*df*: difference between the *df* of the i-th model with the *df* of the null model.

**Table 3:** Summary of coefficients included in the estimated logistic regression models.

Model	Coefficients *	B (SE)	Odds Ratio	95% CI OR
DTK	Constant	−5.93 (1.71)		
	DTK	0.31 (0.08)	1.37	1.15, 1.62
MSF	Constant	−12.48 (3.83)		
	Plank	0.03 (0.01)	1.03	1.01, 1.06
	Handgrip	0.26 (0.10)	1.30	1.06, 1.60
FMC	Constant	23.31 (9.28)		
	T&C	0.46 (0.24)	1.58	1.00, 2.50
	STS&Go	−3.07 (1.10)	0.02	0.003, 0.22
FF + DTK	Constant	−7.91 (3.37)		
	DTK	0.41 (0.17)	1.50	1.07, 2.12
	FFIndex	1.84 (0.65)	6.32	1.76, 22.72

Note: * Coefficients are included only for significant predictors. B: unstandardized beta coefficient; SE: standard error; 95% CI: 95% confidence interval.

## Data Availability

All relevant data are contained in the article. The questionnaire used to assess the Declarative Tactical Knowledge is available upon request to the corresponding author of the study.
